# Complete genome sequence of *Cellulomonas* sp. strain ATA003

**DOI:** 10.1128/mra.01037-23

**Published:** 2024-05-02

**Authors:** Alexander Bartholomäus, Julia Mitzscherling, Daniel Lipus, Dirk Wagner, Paris Lavin, Roberto Contreras, Rómulo Oses

**Affiliations:** 1 GFZ German Research Centre for Geosciences, Section Geomicrobiology, Potsdam, Germany; 2 University of Potsdam, Institute of Geoscience, Potsdam, Germany; 3 Departamento de Biotecnología, Facultad de Ciencias del Mar y Recursos Biológicos, Universidad de Antofagasta, Antofagasta, Chile; 4 Centro Regional de Investigación y Desarrollo Sustentable de Atacama (CRIDESAT), Universidad de Atacama, Copiapó, Chile; University of Strathclyde, Glasgow, United Kingdom

**Keywords:** endophytes, genomes

## Abstract

The Gram-positive, rod-shaped endophytic bacterium *Cellulomonas* sp. strain ATA003 was isolated from the endemic cactus *Maihueniopsis domeykoensis* seeds collected in the Coastal Atacama Desert, Chile. Here, we present a circular genome with a size of 4,084,881 bp and a GC content of 73.8% obtained by Nanopore sequencing.

## ANNOUNCEMENT

The Atacama Desert of northern Chile is an extreme environment characterized by poor soils with low levels of organic matter and nutrients, high-temperature oscillations, UV radiation, and very low annual precipitations. However, the Atacama Desert harbors a rich microbial diversity that has only recently been discovered (1). Here, we identified a seed endophyte (SE) from the Atacama Desert. SEs can colonize endospheric compartments of seeds, providing benefits to host plants such as growth-promoting activities, improved seed germination, or resistance against stresses (2, 3). They are of potential interest for agriculture and medicine (4, 5).

Seeds of *Maihueniopsis domeykoensis* were collected by AELA SpA at Domeyko (28.952722 S, 70.894150 W) and were supplied by the Forestry Institute – INFOR Chile (La Serena, Chile). The surface of the seeds was sterilized by sequential washing in 50% ethanol for 1 min, 2% sodium hypochlorite for 3 min, and 50% ethanol for 30 s, and was rinsed twice with sterile distilled water, followed by grinding the seeds in a clean mortar and suspending in sterile phosphate buffer. The slurry was diluted and spread on “Combined carbon or Rennie semisolid N-free” medium (6) for 24 h at 30°C. After transfer to N-free agar (NFA) and 72 h of incubation at 30°C, individual bacterial colonies were selected and purified by restreaking five times on fresh NFA. All incubation and culturing steps took place under aerobic conditions. Genomic DNA was extracted from a pure culture grown on LB medium at 28°C using the UltraClean microbial DNA isolation kit (MoBio, Carlsbad, CA, USA).

High-molecular-weight DNA was prepared without specific size selection using the rapid sequencing kit SQK-RAD004 (Oxford Nanopore Technologies [ONT], Oxford, UK). The DNA was cleaned using AMPure XP beads (Beckman Coulter, Pasadena, CA, USA). The library was sequenced using the MinION device and the Flongle flow-cell R9.4.1 (ONT). The sequencing ran for 48 h. Default parameters were used for all software unless otherwise specified. The raw sequencing data were base called and demultiplexed with super high accuracy using Guppy v6.0.6 (ONT), resulting in 54,226 reads with an N50 value of 7,051 bp. No further read quality control, trimming, or correction was done. Assembly, polishing, and circularity assessment were performed using Flye v2.9.1 (7) (parameters –meta –nano-raw). The genome was used as-is from Flye without further rotation or flipping of the genome. The genome quality was assessed, and full-length 16S rRNA sequences were recovered using CheckM v1.2.1 (8).

The resulting circular genome was 4,084,881 bp with a GC content of 73.8% and a coverage of 52x. The genome was found to be 69.4% complete and 2.4% contaminated using the Cellulomonas marker set of CheckM v1.2.2 (8). The taxonomic affiliation and closest related species were inferred using GTDB-Tk v2.1.0 (9) for the genome and NCBI BLAST (10) (v2.14.0, nr/nt database) for 16S rRNA. The closest species were *Cellulomonas cellasea* strain NBRC 3753 (ANI 80.35%, accession GCF_006538745.1) based on the genome and *Cellulomonas fimi* strain NCTC7547 (97.65% identity and 100% query coverage, accession LR134387.1) based on 16S rRNA sequence. Gene annotation was performed automatically by NCBI using the PGAP pipeline v6.6 (11).

## Data Availability

The genome of *Cellulomonas* sp. ATA003 was deposited at GenBank under the accession number GCF_031582905.1 and the BioProject accession number PRJNA1010543. The raw reads are accessible via the SRA accession number SRX21545741. The 16S full-length rRNA is also available on GenBank under accession number OR498076.
